# *Pseudomonas aeruginosa* Contact-Dependent Growth Inhibition Plays Dual Role in Host-Pathogen Interactions

**DOI:** 10.1128/mSphere.00336-17

**Published:** 2017-11-15

**Authors:** Jeffrey A. Melvin, Jordan R. Gaston, Shawn N. Phillips, Michael J. Springer, Christopher W. Marshall, Robert M. Q. Shanks, Jennifer M. Bomberger

**Affiliations:** a Department of Microbiology and Molecular Genetics, University of Pittsburgh School of Medicine, Pittsburgh, Pennsylvania, USA; b Department of Ophthalmology, Charles T. Campbell Laboratory of Ophthalmic Microbiology, University of Pittsburgh, Pittsburgh, Pennsylvania, USA; University of Kentucky

**Keywords:** *Pseudomonas aeruginosa*, contact-dependent growth inhibition, toxin/antitoxin systems, virulence determinants

## Abstract

Microorganisms exist in a diverse ecosystem and have evolved many different mechanisms for sensing and influencing the polymicrobial environment around them, utilizing both diffusible and contact-dependent signals. Contact-dependent growth inhibition (CDI) is one such communication system employed by Gram-negative bacteria. In addition to CDI mediation of growth inhibition, recent studies have demonstrated CDI-mediated control of communal behaviors such as biofilm formation. We postulated that CDI may therefore play an active role in host-pathogen interactions, allowing invading strains to establish themselves at polymicrobial mucosal interfaces through competitive interactions while simultaneously facilitating pathogenic capabilities via CDI-mediated signaling. Here, we show that *Pseudomonas aeruginosa* produces two CDI systems capable of mediating competition under conditions of growth on a surface or in liquid. Furthermore, we demonstrated a novel role for these systems in contributing to virulence in acute infection models, likely via posttranscriptional regulation of beneficial behaviors. While we did not observe any role for the *P. aeruginosa* CDI systems in biofilm biogenesis, we did identify for the first time robust CDI-mediated competition during interaction with a mammalian host using a model of chronic respiratory tract infection, as well as evidence that CDI expression is maintained in chronic lung infections. These findings reveal a previously unappreciated role for CDI in host-pathogen interactions and emphasize their importance during infection.

**IMPORTANCE** How bacteria compete and communicate with each other is an increasingly recognized aspect of microbial pathogenesis with a major impact on disease outcomes. Gram-negative bacteria have recently been shown to employ a contact-dependent toxin-antitoxin system to achieve both competition and regulation of their physiology. Here, we show that this system is vital for virulence in acute infection as well as for establishment of chronic infection in the multidrug-resistant pathogen *Pseudomonas aeruginosa*. Greater understanding of the mechanisms underlying bacterial virulence and infection is important for the development of effective therapeutics in the era of increasing antimicrobial resistance.

## Abstract (precis)

How bacteria compete and communicate with each other is an increasingly recognized aspect of microbial pathogenesis with a major impact on disease outcomes. Gram-negative bacteria have recently been shown to employ a contact-dependent toxin-antitoxin system to achieve both competition and regulation of their physiology. Here, we show that this system is vital for virulence in acute infection as well as for establishment of chronic infection in the multidrug-resistant pathogen *Pseudomonas aeruginosa*. Greater understanding of the mechanisms underlying bacterial virulence and infection is important for the development of effective therapeutics in the era of increasing antimicrobial resistance.

## INTRODUCTION

Bacteria both respond to and deploy systems to influence their polymicrobial environment, and the manners in which they interact can have major medical implications for disease progression and treatment efficacy (1, 2). To sense and influence their surrounding microbial community, they utilize a wide array of diffusible signals, including quorum sensing molecules and antimicrobials and contact-dependent systems, such as type 6 secretion systems (T6SS) and contact-dependent growth inhibition (CDI) (3). While T6SS competition and virulence activities have been more extensively characterized, CDI is becoming increasingly recognized as an important mechanism by which many Gram-negative bacteria can mediate both competitive and cooperative interactions (4, 5).

CDI is mediated by two-partner secretion (TPS) systems that consist of an outer membrane pore (CdiB), a toxin-tipped exoprotein that is secreted through the pore (CdiA), and a cytoplasmic antitoxin immunity protein that protects the bacteria against autoinhibition (CdiI). Previous studies in *Escherichia coli*, *Neisseria meningitidis*, *Burkholderia thailandensis*, and other species have demonstrated the ability of nuclease-containing CdiA proteins to inhibit growth in closely related species or strains lacking cognate immunity proteins (4, 6, 7). More recently, work in *Burkholderia* spp. has revealed a role for both CDI-mediated growth inhibition and CDI-mediated signaling in influencing community behaviors such as biofilm formation (5, 6). Confirmation of a role for CDI in host-pathogen interactions, however, is largely limited to a report of low levels of CDI-mediated competition for *Dickeya didantii* on a plant leaf (4).

*Pseudomonas aeruginosa* is a Gram-negative, opportunistic pathogen and etiologic agent associated with disease in both acute and chronic respiratory tract infections, chronic wounds, and infections of medical devices (8). *P. aeruginosa* is subject to both intra- and interspecies interactions within the diverse microbial communities that it occupies, both in the environment and in the context of a host microbiome during infection. A recent study demonstrated the presence of two related CDI systems in *P. aeruginosa* PAO1 and their ability to mediate competition in laboratory media (9). Genes *P. aeruginosa* 0040 (PA0040) and PA0041 and the small open reading frame immediately downstream comprise the system that we refer to as CDI1 (consisting of *cdi1BAI*), and genes PA2463-PA2462 and the small open reading frame immediately downstream comprise the system we refer to as CDI2 (consisting of *cdi2BAI*). The CDI operons share remarkable similarity, as the CdiB proteins and the N-terminal structural shaft region of the CdiA proteins share 97% and 80% identity, respectively, although Cdi2A contains more structural repeats and thus likely produces a longer shaft and potentially different receptor specificity (9, 10). In contrast, the putative RNase toxins on the C terminus of the CdiA proteins and the CdiI immunity proteins share only 26% and 25% identity, respectively. Cdi1A contains a C-terminal putative Mg^2+^-dependent RNase that is homologous to that encoded by the *B. pseudomallei* 1026b CDI_II_ system (11), and Cdi2A contains a C-terminal putative EndoU class RNase that is homologous to that encoded by the *N. meningitidis* NEM8013 MafB_MGI-1_ system (7).

Due to the demonstrated role of CDI in competition and regulation of beneficial behaviors in other species in *in vitro* laboratory environments, we postulated that the *P. aeruginosa* CDI systems would play a role in host-pathogen interactions. Using acute and chronic infection models, we found a role for both CDI-mediated competition and CDI-mediated signaling in pathogenesis. This study for the first time indicates that CDI systems are integral to the infection biology of Gram-negative bacteria.

## RESULTS AND DISCUSSION

**FIG 1:**
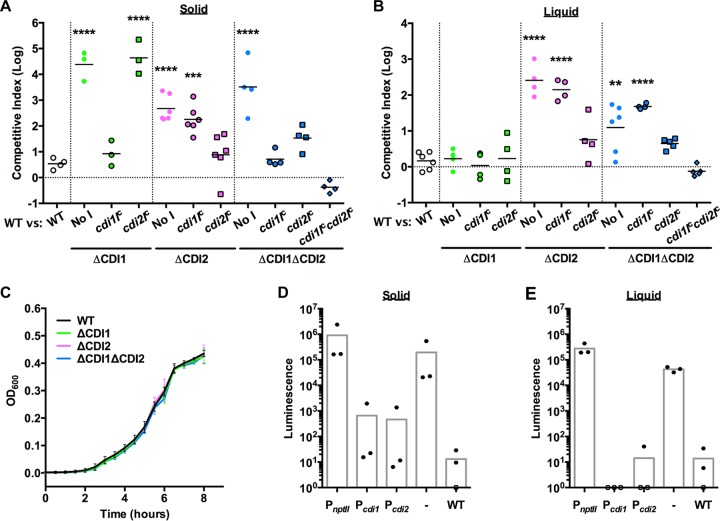
*P. aeruginosa* contains two contact-dependent growth inhibition systems. (A and B) Competitive index of the WT strain versus the target strain designated on the *x* axis on LB agar after 24 h at ambient temperature (A) or in LB broth after 24 h at 37°C (B). "No I" designates a strain with the constitutive P_*nptII*_ promoter without any immunity gene. Each point represents a separate experiment; statistical comparisons correspond to the WT-versus-WT competition. (C) Growth curve in LB broth at 37°C. *n =* 3, points represent the means, and error bars represent standard deviations. (D and E) Luminescence of strains containing a promoter fusion to the *lux* operon inserted on the genome when grown on LB agar at ambient temperature (D) or in LB broth at 37°C (E). The WT designation represents the parent strain without the *lux* operon insertion; “-” indicates a strain containing the *lux* operon without a promoter inserted. Each point represents a separate experiment. Averages represent means. **, *P* < 0.01; ***, *P* < 0.001; ****, *P* < 0.0001.

Through competition experiments using strains with markerless deletion mutations, we measured the ability of the putative *P. aeruginosa* CDI operons to mediate competition on laboratory media, with the wild-type (WT) parent strain outcompeting the mutant strains by 100-fold to 50,000-fold (Fig. 1A and B). Of note, all competitions in this study were performed in an otherwise WT background with an input ratio of 1:1 for inhibitor and target strains. CDI1 was able to mediate competition only on an agar surface, while CDI2 was able to mediate competition both on agar and in broth (Fig. 1A and B). Neither deletion mutation resulted in defects in its growth rate in LB (Fig. 1C) or in minimal medium (see Fig. S1 in the supplemental material), and both were protected only by expression of the cognate *cdiI* gene (Fig. 1A and B), indicating that the competitive advantage of the WT strain was due to the lack of the cognate CdiI immunity protein in the deletion mutant strains. It is likely that the growth inhibition activity is due to intracellular RNase activity in the bacteria lacking an immunity protein.

10.1128/mSphere.00336-17.1 FIG S1  Growth of CDI mutant strains is not altered in minimal medium. Data represent growth curve in M9 minimal medium at 37°C. *n =* 3, points represent the means, and error bars represent standard deviations. Download FIG S1, PDF file, 0.1 MB. Copyright © 2017 Melvin et al. 2017 Melvin et al. This content is distributed under the terms of the Creative Commons Attribution 4.0 International license.

The differences in the ability of the CDI systems to mediate competition in suspension or on a surface did not appear to be due to differential levels of expression of the operons under the different conditions (Fig. 1D and E). Regulation is likely achieved by a repressor, as a promoterless luciferase fusion strain displayed far more luminescence than the CDI promoter fusion strains. Additionally, the *P. aeruginosa* CDI systems did not appear to display stochastic regulation such as has been described for other species (6), as no blue colonies were observed when over 1,000 CDI promoter-*lacZ* fusion strain colonies were grown on X-Gal (5-bromo-4-chloro-3-indolyl-β-d-galactopyranoside)-containing media (Fig. S2). Similarly to other CDI systems (4, 6), both *P. aeruginosa* CDI operons were potent growth inhibitors even though they are tightly regulated.

10.1128/mSphere.00336-17.2 FIG S2  *P. aeruginosa* CDI promoters repress LacZ production. *lacZ* reporter fusion strains were plated on LB agar containing X-Gal. Images are representative of experiments conducted 3 times. Download FIG S2, PDF file, 1.6 MB. Copyright © 2017 Melvin et al. 2017 Melvin et al. This content is distributed under the terms of the Creative Commons Attribution 4.0 International license.

**FIG 2:**
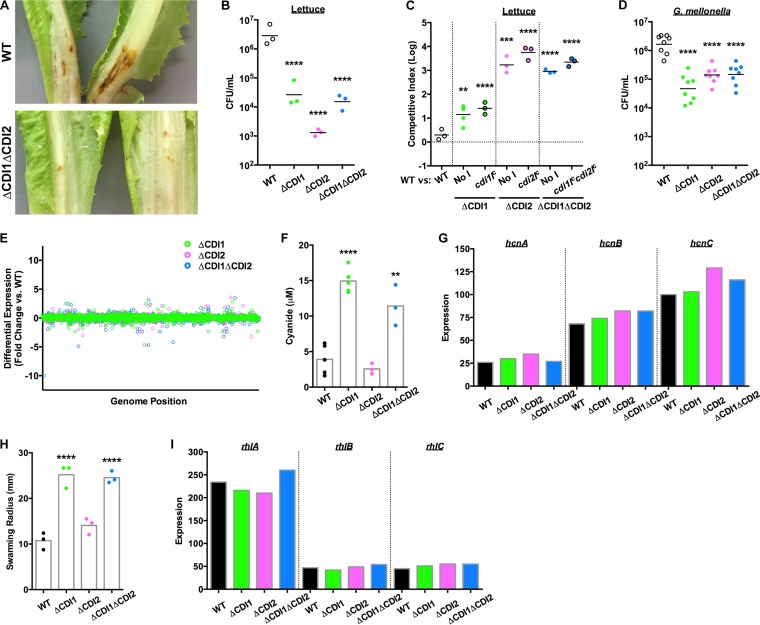
*P. aeruginosa* CDI systems mediate posttranscriptional regulation of virulence. (A) Tissue maceration of lettuce after 7 days at ambient temperature. (B) *P. aeruginosa* burden in lettuce infection sites after 3 days at ambient temperature. Each point represents a separate head of lettuce; data represent results of statistical comparisons to the WT strain. (C) Competitive index of the WT strain versus the target strain designated on the *x* axis in lettuce after 3 days at ambient temperature. "No I" designates a strain with the constitutive P_*nptII*_ promoter without any immunity gene. Each point represents a separate head of lettuce; data represent results of statistical comparisons corresponding to the WT-versus-WT competition. (D) *P. aeruginosa* burden in *G. mellonella* larvae after 1 day at ambient temperature. Each point represents a separate larva; data represent results of statistical comparisons to the WT strain. (E) Differential expression of genes in the CDI mutant strains compared to the WT strain. (F) Cyanide production of the WT and CDI mutant strains. Each point represents a separate experiment; data represent results of statistical comparisons to the WT strain. (G) Expression values of cyanide synthesis operon in the WT and CDI mutant strains, as calculated by Rockhopper. (H) Quantification of swarming distance for the WT and CDI mutant strains after 24 h at ambient temperature. Each point represents a separate experiment; data represent results of statistical comparisons to the WT strain. (I) Expression values of rhamnolipid synthesis operon in the WT and CDI mutant strains, as calculated by Rockhopper. For RNA-seq expression data, *n =* 2 and each point represents a weighted average. For all other data, averages represent the means. **, *P* < 0.01; ***, *P* < 0.001; ****, *P* < 0.0001.

Acute *P. aeruginosa* infections are among the most severe nosocomial infections (8), necessitating a better understanding of the mechanisms underlying these disease states. Transposon mutagenesis studies have suggested that the *P. aeruginosa* CDI systems may contribute to virulence in acute models of infection (12–14). We found that *P. aeruginosa* strains lacking the CDI systems had virulence defects in a lettuce model of acute infection, in terms of both tissue maceration and *P. aeruginosa* burden (Fig. 2A and B). In mixed infections, CDI mutant strains expressing cognate *cdiI* genes were not rescued during coinfection with the WT strain (Fig. 2C), indicating that the virulence defect was independent of CDI-mediated competition. Similarly, the CDI mutant strains displayed a defect in bacterial burden in a waxworm moth (*Galleria mellonella*) larva model of acute infection (Fig. 2D). Combined, these data indicate that *P. aeruginosa* CDI modulates virulence in a noncompetitive manner. Previous work has described transcriptional differences mediated by CDI that modulate biofilm formation in *Burkholderia thailandensis* (5), so we characterized the transcriptomes of the CDI mutant strains to determine whether CDI-mediated transcriptional regulation was responsible for the observed virulence defects. However, very few genes showed statistically significant differential expression compared to those carried by the WT strain (Fig. 2E; see also Table S1 in the supplemental material). In contrast, we observed phenotypic changes in the CDI mutant strains, including cyanide production and swarming motility changes (Fig. 2F and H, respectively). These changes were not reflected in the transcriptomic data (Fig. 2G and I), suggesting that the phenotypic differences were the result of posttranscriptional regulation. While we observed cyanide and swarming phenotypes in the strains lacking the CDI1 system, changes in cyanide production or swarming motility likely do not represent the mechanism behind the observed virulence defect, as strains lacking the CDI2 system were equally deficient in the infection models and did not produce the same changes in cyanide production or swarming motility. Rather, we interpret these combined data as revealing a general phenomenon of posttranscriptional regulation by CDI that results in as-yet-unidentified changes that detrimentally affect the virulence of *P. aeruginosa* during acute infection. Additionally, these data suggest that, while some of the regulatory activities of the CDI1 and CDI2 systems may overlap, resulting in the observed virulence defects, there are clearly distinct regulatory functions as well. Of note, when the strain lacking CDI1 (and constitutively expressing *cdi1I*) was grown in contact with WT bacteria, the level of cyanide production was still elevated (Fig. S3). Since the WT bacteria were unable to reduce cyanide production by the ΔCDI1 bacteria, these data indicate that CDI-mediated regulation of cyanide production is facilitated not by intercellular intoxication but rather via an intracellular response to the presence or absence of the CDI1 system.

10.1128/mSphere.00336-17.3 FIG S3  CDI-mediated regulation of cyanide production is not due to intercellular signaling. Data represent cyanide production of the WT and ΔCDI1 mutant strains grown separately and together. Each point represents a separate experiment; averages represent the means. Data represent results of statistical comparisons to the WT strain. *, *P* < 0.05; **, *P* < 0.01; ***, *P* < 0.001. Download FIG S3, PDF file, 0.3 MB. Copyright © 2017 Melvin et al. 2017 Melvin et al. This content is distributed under the terms of the Creative Commons Attribution 4.0 International license.

10.1128/mSphere.00336-17.8 TABLE S1  Significantly different levels of gene expression in CDI mutant strains. Download TABLE S1, DOCX file, 0.02 MB. Copyright © 2017 Melvin et al. 2017 Melvin et al. This content is distributed under the terms of the Creative Commons Attribution 4.0 International license.

**FIG 3:**
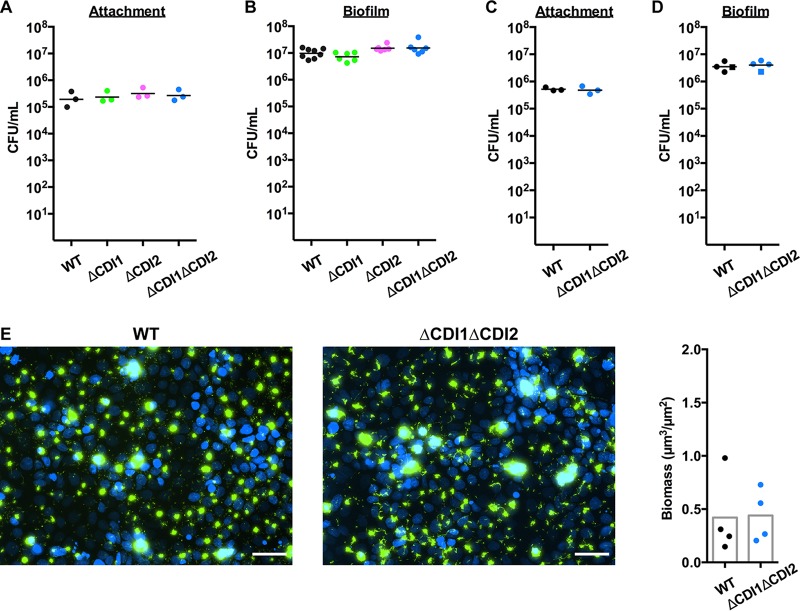
*P. aeruginosa* CDI systems do not modulate biofilm biogenesis on respiratory epithelium. (A) Attachment of WT and CDI mutant strains to differentiated CF bronchial epithelium after 1 h at 37°C. (B) Biofilm formation of WT and CDI mutant strains on differentiated CF bronchial epithelium after 6 h at 37°C. (C) Attachment of WT and ΔCDI1ΔCDI2 strains to primary differentiated non-CF bronchial epithelium after 1 h at 37°C. (D) Biofilm formation of WT and ΔCDI1ΔCDI2 strains on primary differentiated non-CF bronchial epithelium after 6 h at 37°C. Different symbols represent cells from different subjects. Each point represents a separate experiment for the bronchial epithelium interaction experiments. (E) Images of WT and ΔCDI1ΔCDI2 strains producing GFP inoculated on differentiated CF bronchial epithelium under conditions of perfusion after 6 h at 37°C. Blue, epithelial cell nuclei; green, *P. aeruginosa*. Scale bar, 50 μm. Biofilm biomass quantification is graphed to the right; each point represents an individual experiment. Averages represent the means.

One of the major phenotypes associated with CDI-mediated regulation in *Burkholderia* spp. is biofilm formation (15), and *P. aeruginosa* utilizes biofilms in a wide variety of chronic infections to devastating effect (16). To investigate whether CDI mediates biofilm biogenesis in *P. aeruginosa*, we first assessed the role of CDI in aggregation and biofilm formation on abiotic surfaces. While we detected minor defects in aggregation and surface attachment (Fig. S4), overall biofilm formation on a variety of abiotic surfaces and mediums was unperturbed in the strains lacking the CDI systems (Fig. S5). To investigate biofilm biogenesis on a more relevant substrate and to investigate the possibility that the *P. aeruginosa* CDI systems play a role during chronic infection, we utilized specialized models of chronic infection in the respiratory tract (17). In these models, *P. aeruginosa* rapidly forms 10-to-50-μm-diameter aggregates that are similar in appearance to those found in sputum from cystic fibrosis (CF) patients (18) and display the hallmarks of mature biofilms, including transcriptional changes associated with biofilm growth, polysaccharide and quorum sensing molecule production, and vastly increased antibiotic resistance (17, 19). We found that the *P. aeruginosa* CDI systems were not required for attachment to or biofilm biogenesis on differentiated CF bronchial epithelial cells (Fig. 3A and B). Likewise, a strain lacking both CDI systems had no defects in attachment to or biofilm formation on primary differentiated non-CF bronchial epithelial cells (Fig. 3C and D). Another *P. aeruginosa* TPS exoprotein, CdrA, was discovered to be necessary for biofilm formation under conditions of shear stress (20), so we used a live-cell imaging assay to quantify biofilm formation on differentiated CF bronchial epithelial cells under conditions of perfusion (17), finding that there was also no difference in biofilm biogenesis under these conditions (Fig. 3E). Combined, these data indicate that, while the *P. aeruginosa* CDI systems mediate virulence activities in acute models of infection, they do not appear to play a major role in regulating behaviors required for initiation of chronic infections.

10.1128/mSphere.00336-17.4 FIG S4  *P. aeruginosa* CDI systems contribute to bacterial aggregation via protein-protein interactions and adherence to abiotic surfaces. (A) Aggregation of WT and CDI mutant strains over time. Data represent results of statistical comparisons to the WT strain at each time point. *n =* 7; points represent the means; error bars represent standard deviations; data represent results of statistical comparisons to the WT strain. (B) Hydrophobicity assay assessing the ability of bacteria to segregate to a hydrophobic phase. Data represent the optical density of WT and CDI mutant strains in aqueous phase before and after mixing with hydrophobic phase. *n =* 4; points represent the means; error bars represent standard deviations; data represent results of statistical comparisons to the WT strain. (C) Representative images of WT and CDI mutant strains attached to glass coverslips after 1 h at ambient temperature. Quantification of attached bacteria is graphed to the right. Averages represent the means; statistical comparisons are indicated. *, *P* < 0.05; ***, *P* < 0.001. Download FIG S4, PDF file, 1.1 MB. Copyright © 2017 Melvin et al. 2017 Melvin et al. This content is distributed under the terms of the Creative Commons Attribution 4.0 International license.

10.1128/mSphere.00336-17.5 FIG S5  *P. aeruginosa* CDI systems do not contribute to biofilm formation on abiotic surfaces. (A) Images of WT and CDI mutant strain biofilms formed on coverslips after 24 h and 72 h at ambient temperature. (B) Crystal violet quantification of WT and CDI mutant strain biofilms formed on vinyl 96-well plates after 24 h at 37°C. Biofilms were grown in M9 minimal medium (top; image of biofilms shown above), M63 minimal medium (middle), or LB (bottom). Averages represent the means. Download FIG S5, PDF file, 1.5 MB. Copyright © 2017 Melvin et al. 2017 Melvin et al. This content is distributed under the terms of the Creative Commons Attribution 4.0 International license.

**FIG 4:**
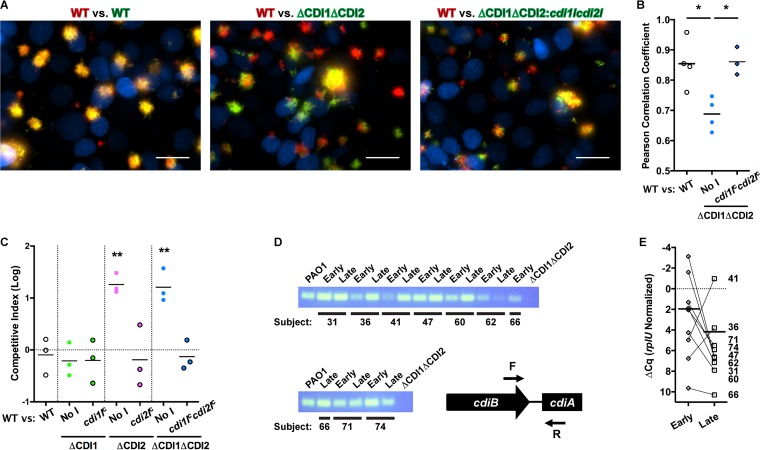
*P. aeruginosa* CDI systems mediate competition during biofilm biogenesis on respiratory epithelium. (A) Images of WT and ΔCDI1ΔCDI2 strains coinoculated on differentiated CF bronchial epithelium under perfusion after 6 h at 37°C. Blue, epithelial cell nuclei; red, WT *P. aeruginosa*; green, WT, ΔCDI1ΔCDI2, or ΔCDI1ΔCDI2::*cdi1I*^c^*cdi2I*^c^ *P. aeruginosa*. Scale bar, 50 μm. (B) Analysis of colocalization of the WT strain with the various competitor strains shown in panel A. Each point represents a separate experiment; statistical comparisons are shown. (C) Competitive index of the WT strain versus the target strain designated on the *x* axis on respiratory epithelium after 6 h at 37°C. "No I" designates a strain with the constitutive P_*nptII*_ promoter without any immunity gene. Each point represents a separate experiment; statistical comparisons correspond to the WT-versus-WT competition. (D) Image of agarose gel for PCR analysis performed for genomic DNA from longitudinally paired early-late *P. aeruginosa* isolates from pediatric CF lungs to detect the presence of a CDI operon. The location and orientation of the primer set are shown at bottom right. (E) Relative levels of expression of the CDI operon in paired isolates grown in LB broth at 37°C. The axis is inverted to account for the fact that a lower Δ*C*_*q*_ value corresponds to higher expression. Each point represents a separate isolate. *, *P* < 0.05; **, *P* < 0.01.

While we were unable to detect a contribution of *P. aeruginosa* CDI systems to biofilm biogenesis, we investigated whether the CDI systems were instead functioning in a competitive manner during the initial stages of colonization of the respiratory epithelium. Using our live-cell imaging assay of biofilm formation on the CF bronchial epithelium, we found that a strain lacking both CDI systems was excluded from biofilms formed by the WT strain, while the CDI mutant strain complemented with both *cdiI* genes was able to form mixed biofilms with the WT strain just as well as WT strains producing different fluorescent proteins (Fig. 4A). Colocalization analysis revealed this effect to be statistically significant (Fig. 4B). A quantitative competition assay performed on differentiated CF bronchial epithelium showed that the *P. aeruginosa* CDI2 system was capable of mediating competition during biofilm biogenesis on the respiratory epithelium, whereas the CDI1 system was not (Fig. 4C). To ascertain whether the *P. aeruginosa* CDI systems might play a role in colonization of the CF lung, we examined a set of longitudinally paired isolates from nine pediatric CF patients (21). Using a primer set that is specific for the *cdiB*-*cdiA* boundary and able to detect either system, we performed PCR on genomic DNA (gDNA) from these isolates and found that all of the strains contained at least one CDI system (Fig. 4D). Additionally, quantitative PCR analysis performed on RNA extracted from each strain showed that each isolate, regardless of whether it was early or late, had detectable expression of CDI genes when grown in LB medium (Fig. 4E). This is in agreement with transcriptomics studies showing detectable expression of CDI genes in clinical isolates of *P. aeruginosa* grown in minimal medium (22) and, most importantly, directly from CF sputum (22, 23). Together with the fact that *P. aeruginosa* CDI systems can mediate competition during interaction with the respiratory epithelium, these results suggest that *P. aeruginosa* maintains CDI expression during colonization of the CF respiratory tract to exert dominance over cohabitating strains, in addition to utilizing other well-characterized competitive advantages (8). Along with the likelihood that CDI operon expression is tightly regulated (Fig. 1; see also Fig. S2 and S6), altered CDI operon expression may also be an adaptive change that leads to altered bacterial physiology such as was observed in our acute infection models (Fig. 2). This ability to modulate physiology may contribute to persistence in the chronic pulmonary infection environment in contrast to virulence in the early stages of chronic infection.

10.1128/mSphere.00336-17.6 FIG S6  *P. aeruginosa* CDI promoters repress *lux* operon activity during interaction with eukaryotic hosts. (A) Luminescence and bacterial burden of strains containing a promoter fusion to the *lux* operon inserted on the genome in lettuce infection sites after 3 days at ambient temperature. "MgSO_4_" indicates lettuce inoculated with vehicle; “-” indicates a strain containing the *lux* operon without a promoter inserted. (B) Luminescence and bacterial burden of strains containing a promoter fusion to the *lux* operon inserted on the genome in *G. mellonella* infection after 1 day at ambient temperature. "MgSO_4_" indicates *G. mellonella* inoculated with vehicle; “-” indicates a strain containing the *lux* operon without a promoter inserted. (C) Luminescence and bacterial burden of strains containing a promoter fusion to the *lux* operon inserted on the genome on differentiated CF bronchial epithelium after 6 h at 37°C. "MEM" indicates CF bronchial epithelial cells inoculated with vehicle; “-” indicates a strain containing the *lux* operon without a promoter inserted. Averages represent means; bars represent luminescence; dots represent CFU per milliliter. Download FIG S6, PDF file, 0.3 MB. Copyright © 2017 Melvin et al. 2017 Melvin et al. This content is distributed under the terms of the Creative Commons Attribution 4.0 International license.

Taken together, our data suggest a model where *P. aeruginosa* CDI systems mediate competitive interactions and beneficial behaviors simultaneously during pathogenesis. The CDI systems appear to be quite potent, particularly when employed on a surface, with a WT strain outcompeting strains lacking cognate immunity proteins by 100-fold to 50,000-fold on laboratory media over a 24-h time period. Perhaps more importantly, WT strains outcompeted CDI mutant strains more than 10-fold on respiratory epithelial cells over only a 6-h time period, effectively excluding strains lacking CDI from these epithelium-associated biofilms. Additionally, this competitive advantage was achieved without high levels of expression under any condition tested (Fig. 1; see also Fig. S2 and S6).

While the results of the competitive interactions occurring during an infection are somewhat obvious, the virulence activities are more complex. The only previously identified method of CDI-mediated signal transduction, i.e., that exhibited by the *B. thailandensis* DNase-based CdiA toxin, is via indirect transcriptional regulation (5). In the *B. thailandensis* system, CdiA proteins can transmit nuclease activity-dependent signals to intoxicated neighboring bacteria, a phenomenon termed contact-dependent signaling (5). Here, our findings suggest instead that *P. aeruginosa* CdiA proteins can also exert their effects on the bacterial cells that produce them, as strains lacking the CDI systems displayed defective virulence that was independent of competitive interactions even during coinoculation with WT strains (Fig. 2C). Indeed, previous reports suggested that CdiA ribonucleases maintain some nuclease activity even in the presence of CdiI immunity proteins (24). In support of this model, contact with WT bacteria was unable to restore suppression of cyanide production by bacteria lacking the CDI1 system (Fig. S3), suggesting that CdiA proteins can alter bacterial physiology prior to secretion. While we were able to detect phenotypic changes associated with a lack of the CDI1 system (i.e., increased cyanide production and swarming behavior) and these behaviors can contribute to virulence (14, 25, 26), we do not think that they represent the specific mechanism by which virulence is altered in strains lacking the CDI1 system. Both of these behaviors might be expected to increase bacterial virulence in an acute model of infection, and strains lacking the CDI1 system and the CDI2 system, which did not display altered cyanide production or swarming behavior, had a defect in virulence. Rather, we interpret these data to mean that lack of either CDI system results in as-yet-unknown changes in bacterial physiology that reduce bacterial virulence.

In contrast with the *B. thailandensis* CDI system, it is perhaps not surprising that CDI systems consisting of likely RNases may regulate bacterial physiology directly, by cleaving mRNAs, rRNAs, tRNAs, or posttranscriptional regulator small RNAs. The primary targets identified for CdiA ribonucleases are exposed regions of rRNAs and tRNAs (4, 7, 24, 27, 28), which are not structurally dissimilar from noncoding small RNAs that are known to have broad regulatory activity. It is therefore possible that the *P. aeruginosa* CDI systems may achieve signal transduction via modulation of small-RNA regulatory activity, a theory which is suggested by the finding that the small RNA called P30 (encoded by the PA4726.2 gene) has slightly increased absolute expression levels in strains lacking the CDI2 system (Table S1). Additionally, a previous study demonstrated regulation of the *P. aeruginosa* CDI systems by the posttranscriptional regulator RsmA (9), which itself is regulated by the small RNAs *rsmY* and *rsmZ* (29). It is consequently possible that *P. aeruginosa* CDI systems may feed back to subtly alter activities of *rsmY* and *rsmZ*, which are known regulators of both swarming and cyanide production (30). Intriguingly, the strains lacking CDI1, which displayed detectable phenotypic changes in the absence of obvious transcriptomic variations, had slight though not significant increases in *rsmY* and *rsmZ* expression (Fig. S7). An alternative explanation is that partial degradation of mRNAs, tRNAs, or rRNAs may also be sensed and may result in altered posttranscriptional regulation, potentially via altered translational efficiency. This idea is supported by the fact that strains lacking both CDI1 and CDI2 have increased levels of 16S and 23S rRNAs, which may be indicative of relief of CDI-mediated degradation of these RNAs in the strain lacking both CDI systems (Fig. S7 and Table S1).

10.1128/mSphere.00336-17.7 FIG S7  *rsm* and ribosomal RNA expression is altered in the absence of CDI systems. (A) Expression values of *rsm* small-RNA genes in the WT and CDI mutant strains, as calculated by Rockhopper. (B) Expression values of rRNA genes in the WT and CDI mutant strains, as calculated by Rockhopper. *n =* 2; each point represents the weighted average for a different rRNA gene. *P. aeruginosa* harbors 4 examples of each rRNA gene. Download FIG S7, PDF file, 0.2 MB. Copyright © 2017 Melvin et al. 2017 Melvin et al. This content is distributed under the terms of the Creative Commons Attribution 4.0 International license.

While the exact mechanism has yet to be elucidated, CDI-mediated regulation is required for *P. aeruginosa* virulence during acute infection. Likewise, the ability of CDI to mediate competition during the early stages of chronic infection is formidable. These findings therefore reveal a new avenue of research for CDI systems, which are widespread among Gram-negative bacteria and have been characterized in only a few species. Furthermore, this report provides a rationale for investigating therapeutic targeting of CDI systems in pathogenic bacteria, an important endeavor for treatment of infections by bacteria such as *P. aeruginosa* that are becoming increasingly resistant to all antibiotics that are used clinically. As appreciation of biogeography and ecological niches in microbial infections expands, understanding the mechanisms that microbes use both to establish dominance and to communicate with their polymicrobial environment is becoming increasingly important. The role of CDI systems in host-pathogen interactions is not well understood, and our study results suggest that they mediate early colonization dynamics at infection sites.

## MATERIALS AND METHODS

### Strains and growth conditions.

Bacteria were grown in lysogeny broth (LB; Sigma), M9 (BD Difco), or M63 medium at 37°C, except where indicated otherwise. Genetic alterations were performed in *P. aeruginosa* strain PAO1. Primers, plasmids, and strains are listed in Table S2 in the supplemental material. Deletion mutations were created using the pMQ30 allelic replacement plasmid (31). Briefly, upstream and downstream regions were amplified by PCR, and allelic replacement constructs were inserted into pMQ30 using restriction enzyme digestion and ligation. Ligated plasmids were transformed into DH5α *E. coli*, and bacteria carrying ligated plasmids were selected for on LB agar containing 10 μg/ml gentamicin (Atlanta Biologicals). Plasmids were isolated from *E. coli* by the use of a Miniprep system (Qiagen), and insertions were confirmed by DNA sequencing (Eurofins) and transformed into RHO3 *E. coli* (32). Plasmids were introduced into *P. aeruginosa* by conjugation with RHO3 *E. coli*. Recombination events were selected for on LB agar containing 50 μg/ml gentamicin. Second recombination events were selected for by growing *P. aeruginosa* in LB overnight and plating on LB containing 5% sucrose at room temperature. Deletion mutant strains were identified by PCR, sucrose resistance, and gentamicin resistance. Mutations were confirmed by whole-genome sequencing on a NextSeq 500 system (Illumina) with 2 × 150-bp libraries. Variant calling was performed using breseq version 0.28.1 (33). No additional mutations were found in any of the mutant strains. Antibiotic resistance for competition assays was introduced by electroporation of pUC18-mini-Tn*7* plasmids (6, 34). Complementation was achieved by electroporation of pTNS3 (35) and pUC18-mini-Tn*7* plasmids carrying immunity genes fused to the constitutive promoter P_*nptII*_ which were constructed by PCR amplification of immunity genes, restriction enzyme digestion, and ligation. Construction of reporter plasmids (*lacZ* or *lux* operon transcriptional fusions in pUC18-mini-Tn7 plasmids [34]) was also achieved by PCR amplification of *cdiB* promoter regions, restriction enzyme digestion, and ligation, and the plasmids were introduced by electroporation.

10.1128/mSphere.00336-17.9 TABLE S2  Primers, plasmids, and strains applied in this study. Download TABLE S2, DOCX file, 0.02 MB. Copyright © 2017 Melvin et al. 2017 Melvin et al. This content is distributed under the terms of the Creative Commons Attribution 4.0 International license.

### Mammalian cell culture.

Immortalized human bronchial epithelial cells from a ΔF508 homozygous cystic fibrosis patient (CFBE41o- [36]) were cultured at 37°C and 5% CO_2_ in minimal essential medium (MEM) (Gibco) supplemented with 2 mM l-glutamine, 5 U/ml penicillin, and 5 μg/ml streptomycin (Sigma); 0.5 μg/ml plasmocin prophylactic (InvivoGen); and 10% fetal bovine serum (FBS; Gemini Bio-Products). CFBE41o- cells were seeded at confluence on Transwell permeable membrane supports (Costar) and differentiated at the air-liquid interface for 1 week prior to use or were seeded at confluence and polarized on 40-mm-diameter glass coverslips (Fisher Scientific) for 1 week prior to use. CFBE41o- cells are not on the list of commonly misidentified cells, and cells were tested quarterly for the presence of mycoplasma using a mycoplasma detection kit (Southern Biotech).

Primary human bronchial epithelial cells were obtained from explanted lungs of adult bronchiectasis patients with informed consent under a protocol approved by the Institutional Review Board at the University of Pittsburgh (REN16100171). All relevant ethical regulations were complied with. Epithelial cells were seeded on permeable membrane supports and differentiated at the air-liquid interface prior to use.

### Bioinformatics analysis.

Protein sequences were analyzed using Clustal Omega version 1.2.2 (37). RNase predictions were performed using NCBI BLASTP.

### Competition in growth media.

*P. aeruginosa* cultures grown overnight in LB containing appropriate antibiotics were washed with phosphate-buffered saline (PBS) and diluted to an optical density at 600 (OD_600_) of 0.2 in PBS. Normalized strains were mixed 1:1, and mixtures were grown in LB at 37°C or on LB agar at room temperature for 24 h. Inoculums were serially diluted and spotted in triplicate on LB agar containing 50 μg/ml gentamicin or 50 μg/ml tetracycline. Liquid competitions were serially diluted and spotted in triplicate on LB agar containing 50 μg/ml gentamicin or 50 μg/ml tetracycline, while solid competitions were scraped from edges, resuspended in PBS, serially diluted, and spotted in triplicate on LB agar containing 50 μg/ml gentamicin or 50 μg/ml tetracycline. CFU counts were determined for inoculums and competitions, and competitive indices were calculated as follows: [(WT_output_/competitor_output_)/(WT_input_/competitor_input_)].

### Growth curves.

*P. aeruginosa* cultures grown overnight in LB were washed with LB or M9 and diluted to an OD_600_ of 0.05. Normalized bacteria were diluted 1:100 in fresh media and inoculated in quadruplicate in a 96-well plate. Plates were incubated at 37°C with 3 s of shaking prior to OD_600_ measurement every 30 min on a SpectraMax M2 plate reader (Molecular Devices).

### Promoter activity.

Transcriptional fusion reporter plasmids inserted at a single site on the genome were used to assess promoter activity. *P. aeruginosa* liquid cultures grown overnight in LB containing 50 μg/ml gentamicin were washed with PBS, while colonies from LB agar containing 50 μg/ml gentamicin were resuspended in PBS and subsequently washed with PBS. For luciferase reporter strains, homogenates from infections or PBS suspensions were placed in an opaque 96-well plate and luminescence was measured using a Synergy 2 plate reader (BioTek). β-Galactosidase reporter strains were serially diluted and plated on LB agar containing 50 μg/ml gentamicin and 20 mg/ml X-Gal (Fisher Scientific). Experiments were performed three or more times.

### Lettuce model of acute infection.

*P. aeruginosa* cultures grown overnight in LB were washed with 10 mM MgSO_4_ and diluted to an OD_600_ of 0.2. Normalized bacteria (10 μl) were inoculated into the midrib of romaine lettuce leaves (38). Three leaves were inoculated with each strain and incubated at ambient temperature in humidified containers in the presence of natural light cycling. Plant tissue maceration was observed after 7 days. The experiment was repeated with 3 separate heads of lettuce.

For bacterial burden determinations, two leaves of lettuce were inoculated and infection was allowed to progress as described above for 3 days. For competition experiments, strains were mixed 1:1 prior to inoculation. After 3 days, an approximately 1-cm-by-2-cm piece of lettuce was removed, minced, and placed in a tube with 1-mm-diameter zirconia beads (BioSpec) and 10 mM MgSO_4_ with 0.1% Triton X-100. Lettuce was homogenized on a BeadBeater (BioSpec) for 2 min at maximum beating speed. The homogenate was serially diluted, and 10 µl was spotted in triplicate on LB agar plates containing 50 μg/ml gentamicin or 50 μg/ml tetracycline. CFU counts were determined for inoculums, single-strain infection burdens, and competitions, and competitive indices were calculated for the competitions.

### Insect model of acute infection.

*P. aeruginosa* cultures grown overnight in LB were washed with PBS supplemented with 75 μg/ml ampicillin and diluted to an OD_600_ of 0.5. Normalized bacteria were serially diluted to 10^−5^. Diluted bacteria (10 μl [approximately 25 CFU]) were inoculated behind a rear leg of each chilled *Galleria mellonella* larva (Grubco) used for the assays (39). Eight *G. mellonella* larvae were infected for each strain. Inoculums were confirmed by serial dilution, spot plating, and CFU determination. Infected *G. mellonella* larvae were incubated at ambient temperature for 24 h. After 24 h of infection, *G. mellonella* larvae were punctured and homogenized using 1-mm-diameter zirconia beads (BioSpec)–10 mM MgSO_4_–0.1% Triton X-100 via the use of a BeadBeater with 1-mm-diameter zirconia beads at maximum speed for 2 min. The homogenate was serially diluted, and 10 µl was spotted in triplicate on LB agar plates containing 50 μg/ml gentamicin or 50 μg/ml tetracycline. CFU counts were determined for infection burdens.

### Transcriptomic analysis.

Since both *P. aeruginosa* CDI systems were able to mediate growth inhibition on a solid surface, WT and CDI mutant strains grown overnight in LB were washed in 10 mM MgSO_4_, diluted to an OD_600_ of 1, and spotted on LB agar plates. Plates were incubated at ambient temperature overnight; colony biofilms were then scraped up and resuspended in RNAlater stabilization solution (Invitrogen), and suspensions were stored at 4°C overnight. RNAlater suspensions were diluted with PBS and centrifuged, and bacterial pellets were resuspended in RNA-Bee (AMS Biotechnology) and homogenized with 0.1-mm-diameter zirconia-silica beads on a BeadBeater at maximum beating speed three times for 1 min each time. Chloroform was added, phases were separated by centrifugation, and RNA was precipitated from the aqueous phase by addition of isopropanol in the presence of linear acrylamide (Ambion). Precipitated RNA was collected by centrifugation, and the RNA was washed twice with 75% ethanol. Residual ethanol was removed by centrifugation and drying, and the RNA pellets were resuspended in water. Contaminating DNA was removed using a Turbo DNase kit (Ambion) according to the recommendations of the manufacturer, and RNA was purified using an RNA Clean and Concentrator kit (Zymo Research). Resultant RNA was checked for DNA contamination by PCR analysis for the *rpsL* gene; for RNA integrity by agarose gel analysis to identify 5S, 16S, and 23S bands; and for concentration and purity by the use of a NanoDrop spectrophotometer (Thermo Fisher Scientific). Library preparation and sequencing were performed by the Health Sciences Sequencing Core at Children’s Hospital of Pittsburgh. RNA concentrations were confirmed by fluorometric quantification (Qubit), and the integrity of the results was confirmed by the use of RNA ScreenTape (Agilent). rRNA was removed by the use of a RiboZero Epidemiology rRNA removal kit (Illumina), and sequencing libraries were prepared using a TruSeq Stranded Total RNA Library Prep kit (Illumina). Libraries were sequenced by paired end sequencing on a NextSeq 500 system, collecting approximately 2.5 × 10^7^ reads per sample. Reads were aligned to the *P. aeruginosa* PAO1 genome (>90%) and analyzed using Rockhopper version 2.0.3 (40). False-discovery rate (*q*) values of ≤0.05 were considered significant. Similar results were obtained using CLC Genomics Workbench version 10.0.1 (Qiagen).

### Cyanide production.

*P. aeruginosa* strains were cultured overnight in LB broth at 37°C. Overnight cultures were diluted to an OD_600_ of 2 in LB broth. A 25-µl volume was spread onto a brain heart infusion (BHI; Fisher Scientific) agar plate and incubated overnight at 37°C. Following overnight incubation, 1 ml 4 M NaOH was placed into the inverted lid of each plate, and each plate was placed next to the inverted lid in an airtight plastic container for incubation at 30°C for 4 h (14). Samples and concentration standards were added to 96-well plate wells containing a 1:1 mix of 0.1 M 1,2-dinitrobenzene (Sigma) and 0.2 M 4-nitrobenzaldehyde (Sigma) dissolved in ethylene glycol monomethyl ether (Emsure) and incubated at ambient temperature for 20 min. Absorbance was read from each well at 578 nm. Cyanide concentrations for each sample were then determined based on line fit to the standard curve.

### Swarming motility.

Swarming agar plates consisting of 1.25% Bacto agar, 5 g/liter Casamino Acids, 1 mM MgSO_4_, 0.1 mM CaCl_2_, 200 mM Na_2_HPO_4_, 80 mM K_2_HPO_4_, and 35 mM NaCl (Fisher Scientific) were poured and dried overnight (41). *P. aeruginosa* cultures were grown overnight in LB broth at 37°C. Following overnight incubation, cells were washed in PBS and normalized to an OD_600_ of 4 and 2 µl normalized culture was spotted onto the center of a swarming agar plate and incubated for 24 h at 37°C. After 24 h, the radius of swarming was quantitated at four separate points and the data were averaged.

### Bacterial aggregation.

*P. aeruginosa* cultures were grown overnight in LB broth at 37°C. Following overnight incubation, cells were washed in 10 mM MgSO_4_, and OD_600_ was measured over 4 h.

### Bacterial hydrophobicity.

*P. aeruginosa* cultures were grown overnight in LB broth at 37°C. Following overnight incubation, cells were washed in 10 mM PBS, normalized to an OD_600_ of 1, and mixed with hexadecane (10% final concentration). The reaction mixture was subjected to vortex mixing for 2 min and then allowed to separate for 15 min. The absorbance of the aqueous phase was then measured again (42).

### Attachment to abiotic surface.

*P. aeruginosa* cultures carrying a constitutive *gfp*-expressing plasmid (pSMC21 [17]) grown overnight in LB supplemented with 250 μg/ml carbenicillin (Fisher Scientific) were washed with M9 and diluted to an OD_600_ of 0.5. Normalized bacteria were added to an uncoated 14-mm-diameter no. 0 borosilicate glass bottom microwell dish (MatTek Corporation) and incubated at ambient temperature for 2 h. Bacteria were removed, and attached bacteria were washed with M9. Adherent bacteria were fixed with 2.5% glutaraldehyde–PBS overnight in the dark at 4°C. Fixative was replaced prior to imaging on a Nikon Ti-inverted microscope. Five random fields were collected, and adherent bacteria were quantified using Nikon Elements version 4.30.02 software.

### Biofilm formation on abiotic surfaces.

*P. aeruginosa* cultures grown overnight in LB were normalized by OD_600_. Normalized bacteria were diluted 1:30 in M9, M63, or LB medium and added in triplicate to a 96-well U-bottom plate (Costar). Plates were incubated at 37°C in a humidified bag for 24 h. Wells were washed with water and stained with a 41% crystal violet–12% ethanol–47% water solution. Stained plates were washed with water and imaged. Crystal violet was solubilized with 30% acetic acid and quantified by measuring OD_550_ on a SpectraMax M2 plate reader (43).

*P. aeruginosa* cultures carrying a constitutive *gfp*-expressing plasmid grown overnight in LB supplemented with 250 μg/ml carbenicillin were washed with M9 and diluted to an OD_600_ of 0.5. Normalized bacteria were added to an uncoated 14-mm-diameter no. 0 borosilicate glass bottom microwell dish, and the dish was tilted to create an air-liquid interface on the glass and incubated at ambient temperature in a humidified container for 24 h or 72 h. Bacteria were removed, and bacterial biofilms were washed with M9 and fixed with 2.5% glutaraldehyde–PBS overnight in the dark at 4°C. Fixative was removed, and biofilms were stained with 100 μg/ml tetramethyl rhodamine isocyanate (TRITC)-conjugated Hippeastrum hybrid lectin (HHA; EY Laboratories, Inc.)–PBS for 15 min to visualize Psl polysaccharide (44). Stain was removed, and biofilms were washed prior to imaging on a Nikon Ti-inverted microscope.

### Attachment to respiratory epithelium.

*P. aeruginosa* cultures grown overnight in LB were washed with MEM and diluted to an OD_600_ of 0.5. Normalized bacteria were added to differentiated bronchial epithelial cells at a multiplicity of infection (MOI) of ~25. After 1 h, apical medium was removed and epithelial cells were washed thoroughly with MEM. Attached bacteria were dispersed with MEM containing 0.1% Triton X-100, serially diluted, and spotted on LB agar to determine CFU counts (19).

### Biofilm on respiratory epithelium under static conditions.

*P. aeruginosa* cultures grown overnight in LB were washed with MEM and diluted to an OD_600_ of 0.5. Normalized bacteria were added to differentiated bronchial epithelial cells at an MOI of ~25. After 1 h of attachment, planktonic bacteria were removed and apical medium was replaced with MEM supplemented with 0.4% l-arginine. After 6 h in total, apical medium was removed and epithelial cells were washed with MEM. Biofilms were dispersed with MEM containing 0.1% Triton X-100, serially diluted, and spotted on LB agar to determine CFU counts (19).

### Biofilm on respiratory epithelium under shear stress.

CFBE41o- bronchial epithelial cells were grown as a confluent monolayer for 1 week and stained with Hoescht 33342 (Thermo Fisher Scientific). *P. aeruginosa* cultures carrying a constitutive *gfp*-expressing plasmid grown overnight in LB supplemented with 250 μg/ml carbenicillin were washed with MEM and diluted to an OD_600_ of 0.4. Normalized bacteria were added to epithelial cells in an FCS2 parallel plate flow environmental chamber (Bioptechs). After 2 h of attachment, MEM was perfused over the cells at 20 ml/h. After 6 h in total, biofilms were imaged on a Nikon Ti-inverted microscope. Ten random fields were collected, and biofilm biomass was quantified using Nikon Elements software (45).

### Competition on respiratory epithelium.

*P. aeruginosa* cultures grown overnight in LB were washed with MEM and diluted to an OD_600_ of 0.5. Normalized bacteria were mixed 1:1 for competitions and added to differentiated bronchial epithelial cells at an MOI of ~25. After 1 h of attachment, planktonic bacteria were removed and apical medium was replaced with MEM supplemented with 0.4% l-arginine. After 6 h in total, apical medium was removed and epithelial cells were washed with MEM. Biofilms were dispersed with MEM containing 0.1% Triton X-100 and serially diluted, and 10 µl was spotted in triplicate on LB agar plates containing 50 μg/ml gentamicin or 50 μg/ml tetracycline to determine CFU and to calculate competitive indices.

For live-cell imaging of competitions, WT *P. aeruginosa* cultures carrying a constitutive Td-tomato-producing plasmid (pMQ400) or competitor strains carrying a constitutive *gfp*-expressing plasmid were grown overnight in LB supplemented with 50 μg/ml gentamicin or 250 μg/ml carbenicillin. Bacteria were washed with MEM and diluted to an OD_600_ of 0.4. Normalized bacteria were mixed 1:1 for competitions and added to bronchial epithelial cells that were grown as a confluent monolayer for 1 week and stained with Hoescht 33342. After 2 h of attachment, MEM was perfused over the cells at 20 ml/h. After 6 h in total, biofilms were imaged on a Nikon Ti-inverted microscope. Ten random fields were collected, and colocalization was quantified using Nikon Elements software.

### Detection of CDI operons in CF isolate strains.

Primers were designed that recognized the *cdiB*-*cdiA* junction for both CDI systems in *P. aeruginosa* PAO1 (Table S2). Genomic DNA was isolated from a set of paired longitudinal isolates from pediatric CF patient lungs that were separated in time of collection by 7.7 ± 1.5 years (21) using a DNeasy Blood and Tissue kit (Qiagen). PCR was performed using the primer set, products were electrophoresed on an agarose gel infused with SYBR Safe, and bands were imaged with a UV transilluminator.

### Quantification of CDI operon expression in CF isolate strains.

RNA was isolated from a set of paired longitudinal isolates from CF patient lungs grown overnight in LB broth using the RNA extraction method described in the "Transcriptomic analysis" section. RNA was converted to cDNA using an iScript cDNA synthesis kit (Bio-Rad). Real-time quantitative PCR (RT-qPCR) was performed on a CFX Connect Real-Time PCR detection system (Bio-Rad) using prepared cDNA, primers that recognize both CDI systems from *P. aeruginosa* PAO1 (Table S2), and iQ SYBR green Supermix (Bio-Rad). Quantification cycle (Δ*C*_*q*_) values were calculated for expression of CDI operons compared to *rplU*. Standard deviations in *rplU C*_*q*_ values were less than 10% of the mean *C*_*q*_ value.

### Statistical analyses.

Data were plotted and statistical analyses were performed using Prism version 6.0 software (GraphPad Software, Inc.). For all conditions analyzed, the exact numbers of experiments performed are described in the figure legends. Averages indicated in figures represent means, and error bars represent standard deviations of the means. Statistical significance was determined using ordinary one-way analysis of variance (ANOVA), correcting for multiple comparisons.

### Data availability.

The transcriptome sequencing (RNA-seq) data have been deposited in the NCBI Sequence Read Archive under accession no. SRR6214994 to SRR6214997.

## References

[1] Murray JL, Connell JL, Stacy A, Turner KH, Whiteley M 2014 Mechanisms of synergy in polymicrobial infections. J Microbiol 52:188–199. doi:10.1007/s12275-014-4067-3. 24585050 PMC7090983

[2] Birger RB, Kouyos RD, Cohen T, Griffiths EC, Huijben S, Mina MJ, Volkova V, Grenfell B, Metcalf CJE 2015 The potential impact of coinfection on antimicrobial chemotherapy and drug resistance. Trends Microbiol 23:537–544. doi:10.1016/j.tim.2015.05.002. 26028590 PMC4835347

[3] Phelan VV, Liu WT, Pogliano K, Dorrestein PC 2011 Microbial metabolic exchange—the chemotype-to-phenotype link. Nat Chem Biol 8:26–35. doi:10.1038/nchembio.739. 22173357 PMC3869239

[4] Aoki SK, Diner EJ, de Roodenbeke CT, Burgess BR, Poole SJ, Braaten BA, Jones AM, Webb JS, Hayes CS, Cotter PA, Low DA 2010 A widespread family of polymorphic contact-dependent toxin delivery systems in bacteria. Nature 468:439–442. doi:10.1038/nature09490. 21085179 PMC3058911

[5] Garcia EC, Perault AI, Marlatt SA, Cotter PA 2016 Interbacterial signaling via Burkholderia contact-dependent growth inhibition system proteins. Proc Natl Acad Sci U S A 113:8296–8301. doi:10.1073/pnas.1606323113. 27335458 PMC4961174

[6] Anderson MS, Garcia EC, Cotter PA 2012 The Burkholderia bcpAIOB genes define unique classes of two-partner secretion and contact dependent growth inhibition systems. PLoS Genet 8:e1002877. doi:10.1371/journal.pgen.1002877. 22912595 PMC3415462

[7] Jamet A, Jousset AB, Euphrasie D, Mukorako P, Boucharlat A, Ducousso A, Charbit A, Nassif X 2015 A new family of secreted toxins in pathogenic Neisseria species. PLoS Pathog 11:e1004592. doi:10.1371/journal.ppat.1004592. 25569427 PMC4287609

[8] Juan C, Peña C, Oliver A 2017 Host and pathogen biomarkers for severe Pseudomonas aeruginosa infections. J Infect Dis 215:S44–S51. doi:10.1093/infdis/jiw299. 28375513

[9] Mercy C, Ize B, Salcedo SP, de Bentzmann S, Bigot S 2016 Functional characterization of Pseudomonas contact dependent growth inhibition (CDI) systems. PLoS One 11:e0147435. doi:10.1371/journal.pone.0147435. 26808644 PMC4725963

[10] Ruhe ZC, Nguyen JY, Xiong J, Koskiniemi S, Beck CM, Perkins BR, Low DA, Hayes CS 2017 CdiA effectors use modular receptor-binding domains to recognize target bacteria. mBio 8:e00290-17. doi:10.1128/mBio.00290-17. 28351921 PMC5371414

[11] Morse RP, Nikolakakis KC, Willett JL, Gerrick E, Low DA, Hayes CS, Goulding CW 2012 Structural basis of toxicity and immunity in contact-dependent growth inhibition (CDI) systems. Proc Natl Acad Sci U S A 109:21480–21485. doi:10.1073/pnas.1216238110. 23236156 PMC3535622

[12] Attila C, Ueda A, Cirillo SL, Cirillo JD, Chen W, Wood TK 2008 Pseudomonas aeruginosa PAO1 virulence factors and poplar tree response in the rhizosphere. Microb Biotechnol 1:17–29. doi:10.1111/j.1751-7915.2007.00002.x. 21261818 PMC3864428

[13] Potvin E, Lehoux DE, Kukavica-Ibrulj I, Richard KL, Sanschagrin F, Lau GW, Levesque RC 2003 In vivo functional genomics of Pseudomonas aeruginosa for high-throughput screening of new virulence factors and antibacterial targets. Environ Microbiol 5:1294–1308. doi:10.1046/j.1462-2920.2003.00542.x. 14641575

[14] Gallagher LA, Manoil C 2001 Pseudomonas aeruginosa PAO1 kills Caenorhabditis elegans by cyanide poisoning. J Bacteriol 183:6207–6214. doi:10.1128/JB.183.21.6207-6214.2001. 11591663 PMC100099

[15] Garcia EC, Anderson MS, Hagar JA, Cotter PA 2013 Burkholderia BcpA mediates biofilm formation independently of interbacterial contact-dependent growth inhibition. Mol Microbiol 89:1213–1225. doi:10.1111/mmi.12339. 23879629 PMC3786370

[16] Rybtke MT, Jensen PØ, Høiby N, Givskov M, Tolker-Nielsen T, Bjarnsholt T 2011 The implication of Pseudomonas aeruginosa biofilms in infections. Inflamm Allergy Drug Targets 10:141–157. doi:10.2174/187152811794776222. 21314623

[17] Moreau-Marquis S, Bomberger JM, Anderson GG, Swiatecka-Urban A, Ye S, O’Toole GA, Stanton BA 2008 The DeltaF508-CFTR mutation results in increased biofilm formation by Pseudomonas aeruginosa by increasing iron availability. Am J Physiol Lung Cell Mol Physiol 295:L25–L37. doi:10.1152/ajplung.00391.2007. 18359885 PMC2494796

[18] Bjarnsholt T, Jensen PØ, Fiandaca MJ, Pedersen J, Hansen CR, Andersen CB, Pressler T, Givskov M, Høiby N 2009 Pseudomonas aeruginosa biofilms in the respiratory tract of cystic fibrosis patients. Pediatr Pulmonol 44:547–558. doi:10.1002/ppul.21011. 19418571

[19] Hendricks MR, Lashua LP, Fischer DK, Flitter BA, Eichinger KM, Durbin JE, Sarkar SN, Coyne CB, Empey KM, Bomberger JM 2016 Respiratory syncytial virus infection enhances Pseudomonas aeruginosa biofilm growth through dysregulation of nutritional immunity. Proc Natl Acad Sci U S A 113:1642–1647. doi:10.1073/pnas.1516979113. 26729873 PMC4760822

[20] Borlee BR, Goldman AD, Murakami K, Samudrala R, Wozniak DJ, Parsek MR 2010 Pseudomonas aeruginosa uses a cyclic-di-GMP-regulated adhesin to reinforce the biofilm extracellular matrix. Mol Microbiol 75:827–842. doi:10.1111/j.1365-2958.2009.06991.x. 20088866 PMC2847200

[21] Mulcahy LR, Burns JL, Lory S, Lewis K 2010 Emergence of Pseudomonas aeruginosa strains producing high levels of persister cells in patients with cystic fibrosis. J Bacteriol 192:6191–6199. doi:10.1128/JB.01651-09. 20935098 PMC2981199

[22] Son MS, Matthews WJ Jr., Kang Y, Nguyen DT, Hoang TT 2007 In vivo evidence of Pseudomonas aeruginosa nutrient acquisition and pathogenesis in the lungs of cystic fibrosis patients. Infect Immun 75:5313–5324. doi:10.1128/IAI.01807-06. 17724070 PMC2168270

[23] Lim YW, Schmieder R, Haynes M, Willner D, Furlan M, Youle M, Abbott K, Edwards R, Evangelista J, Conrad D, Rohwer F 2013 Metagenomics and metatranscriptomics: windows on CF-associated viral and microbial communities. J Cyst Fibros 12:154–164. doi:10.1016/j.jcf.2012.07.009. 22951208 PMC3534838

[24] Ruhe ZC, Nguyen JY, Beck CM, Low DA, Hayes CS 2014 The proton motive force is required for translocation of CDI toxins across the inner membrane of target bacteria. Mol Microbiol 94:466–481. doi:10.1111/mmi.12779. 25174572 PMC4191985

[25] Broderick KE, Chan A, Balasubramanian M, Feala J, Reed SL, Panda M, Sharma VS, Pilz RB, Bigby TD, Boss GR 2008 Cyanide produced by human isolates of Pseudomonas aeruginosa contributes to lethality in Drosophila melanogaster. J Infect Dis 197:457–464. doi:10.1086/525282. 18199034

[26] Overhage J, Bains M, Brazas MD, Hancock RE 2008 Swarming of Pseudomonas aeruginosa is a complex adaptation leading to increased production of virulence factors and antibiotic resistance. J Bacteriol 190:2671–2679. doi:10.1128/JB.01659-07. 18245294 PMC2293252

[27] Batot G, Michalska K, Ekberg G, Irimpan EM, Joachimiak G, Jedrzejczak R, Babnigg G, Hayes CS, Joachimiak A, Goulding CW 2017 The CDI toxin of Yersinia kristensenii is a novel bacterial member of the RNase A superfamily. Nucleic Acids Res 45:5013–5025. doi:10.1093/nar/gkx230. 28398546 PMC5435912

[28] Jones AM, Garza-Sánchez F, So J, Hayes CS, Low DA 2017 Activation of contact-dependent antibacterial tRNase toxins by translation elongation factors. Proc Natl Acad Sci U S A 114:E1951–E1957. doi:10.1073/pnas.1619273114. PMC5347540 28223500

[29] Sonnleitner E, Haas D 2011 Small RNAs as regulators of primary and secondary metabolism in Pseudomonas species. Appl Microbiol Biotechnol 91:63–79. doi:10.1007/s00253-011-3332-1. 21607656

[30] Kay E, Humair B, Dénervaud V, Riedel K, Spahr S, Eberl L, Valverde C, Haas D 2006 Two GacA-dependent small RNAs modulate the quorum-sensing response in Pseudomonas aeruginosa. J Bacteriol 188:6026–6033. doi:10.1128/JB.00409-06. 16885472 PMC1540078

[31] Shanks RM, Caiazza NC, Hinsa SM, Toutain CM, O’Toole GA 2006 Saccharomyces cerevisiae-based molecular tool kit for manipulation of genes from gram-negative bacteria. Appl Environ Microbiol 72:5027–5036. doi:10.1128/AEM.00682-06. 16820502 PMC1489352

[32] López CM, Rholl DA, Trunck LA, Schweizer HP 2009 Versatile dual-technology system for markerless allele replacement in Burkholderia pseudomallei. Appl Environ Microbiol 75:6496–6503. doi:10.1128/AEM.01669-09. 19700544 PMC2765137

[33] Deatherage DE, Barrick JE 2014 Identification of mutations in laboratory-evolved microbes from next-generation sequencing data using breseq. Methods Mol Biol 1151:165–188. doi:10.1007/978-1-4939-0554-6_12. 24838886 PMC4239701

[34] Choi KH, Schweizer HP 2006 Mini-Tn7 insertion in bacteria with single attTn7 sites: example Pseudomonas aeruginosa. Nat Protoc 1:153–161. doi:10.1038/nprot.2006.24. 17406227

[35] Choi KH, Mima T, Casart Y, Rholl D, Kumar A, Beacham IR, Schweizer HP 2008 Genetic tools for select-agent-compliant manipulation of Burkholderia pseudomallei. Appl Environ Microbiol 74:1064–1075. doi:10.1128/AEM.02430-07. 18156318 PMC2258562

[36] Cozens AL, Yezzi MJ, Kunzelmann K, Ohrui T, Chin L, Eng K, Finkbeiner WE, Widdicombe JH, Gruenert DC 1994 CFTR expression and chloride secretion in polarized immortal human bronchial epithelial cells. Am J Respir Cell Mol Biol 10:38–47. doi:10.1165/ajrcmb.10.1.7507342. 7507342

[37] Sievers F, Wilm A, Dineen D, Gibson TJ, Karplus K, Li W, Lopez R, McWilliam H, Remmert M, Söding J, Thompson JD, Higgins DG 2011 Fast, scalable generation of high-quality protein multiple sequence alignments using Clustal Omega. Mol Syst Biol 7:539. doi:10.1038/msb.2011.75. 21988835 PMC3261699

[38] Starkey M, Rahme LG 2009 Modeling Pseudomonas aeruginosa pathogenesis in plant hosts. Nat Protoc 4:117–124. doi:10.1038/nprot.2008.224. 19180083 PMC6501584

[39] Ramarao N, Nielsen-Leroux C, Lereclus D 2012 The insect Galleria mellonella as a powerful infection model to investigate bacterial pathogenesis. J Vis Exp 11:e4392. doi:10.3791/4392. PMC3567165 23271509

[40] McClure R, Balasubramanian D, Sun Y, Bobrovskyy M, Sumby P, Genco CA, Vanderpool CK, Tjaden B 2013 Computational analysis of bacterial RNA-Seq data. Nucleic Acids Res 41:e140. doi:10.1093/nar/gkt444. 23716638 PMC3737546

[41] Tremblay J, Déziel E 2008 Improving the reproducibility of Pseudomonas aeruginosa swarming motility assays. J Basic Microbiol 48:509–515. doi:10.1002/jobm.200800030. 18785657

[42] Rosenberg M 1984 Isolation of pigmented and nonpigmented mutants of Serratia marcescens with reduced cell surface hydrophobicity. J Bacteriol 160:480–482. 6384200 10.1128/jb.160.1.480-482.1984 PMC214751

[43] O’Toole GA 2011 Microtiter dish biofilm formation assay. J Vis Exp. doi:10.3791/2437. PMC3182663 21307833

[44] Ma L, Lu H, Sprinkle A, Parsek MR, Wozniak DJ 2007 Pseudomonas aeruginosa Psl is a galactose- and mannose-rich exopolysaccharide. J Bacteriol 189:8353–8356. doi:10.1128/JB.00620-07. 17631634 PMC2168683

[45] Melvin JA, Lashua LP, Kiedrowski MR, Yang G, Deslouches B, Montelaro RC, Bomberger JM 2016 Simultaneous antibiofilm and antiviral activities of an engineered antimicrobial peptide during virus-bacterium coinfection. mSphere 189:e00083-16. doi:10.1128/mSphere.00083-16. 27303744 PMC4888888

