# Supplementary figures and images for: Pseudomonas aeruginosa Contact-Dependent Growth Inhibition Plays Dual Role in Host-Pathogen Interactions
Source: mSphere. 2017 Nov 15;2(6):e00336-17. doi: 10.1128/mSphere.00336-17 (PMC5687917; doi:10.1128/mSphere.00336-17)

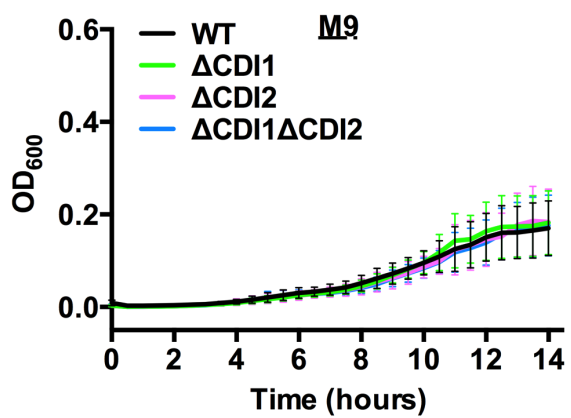

Supplement: FIG S1 [file sph006172403sf1.pdf]

No promoter

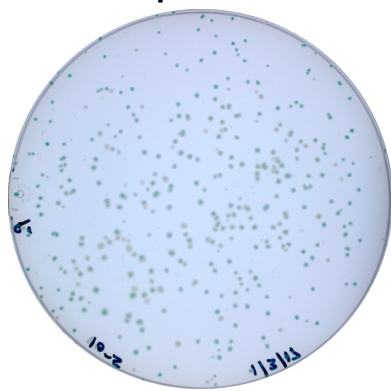

Constitutive promoter

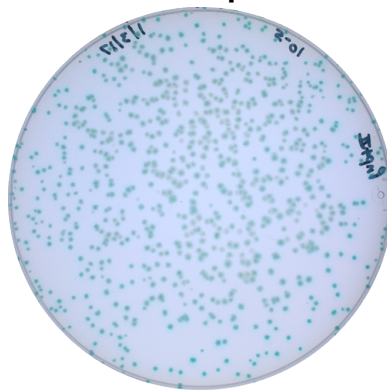

**P**<sub>*cdi1*</sub>

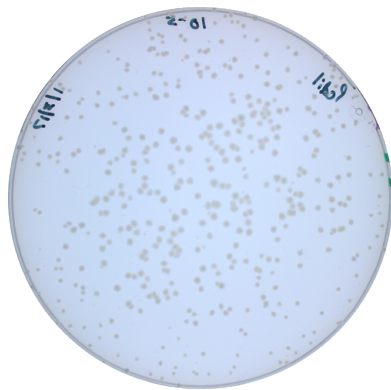

**P**<sub>*cdi2*</sub>

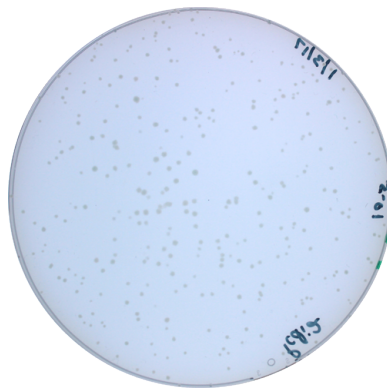

Supplement: FIG S2 [file sph006172403sf2.pdf]

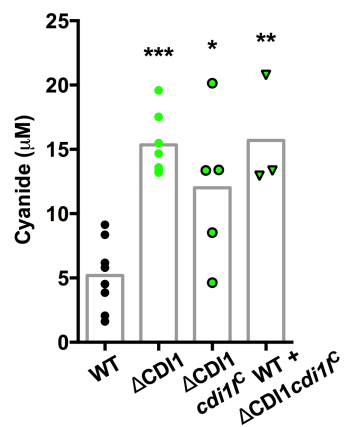

Supplement: FIG S3 [file sph006172403sf3.pdf]

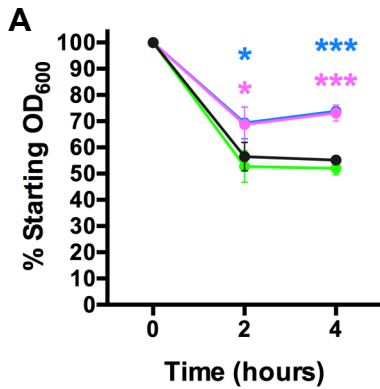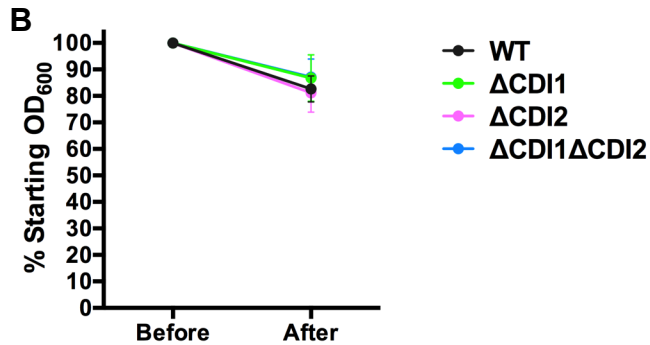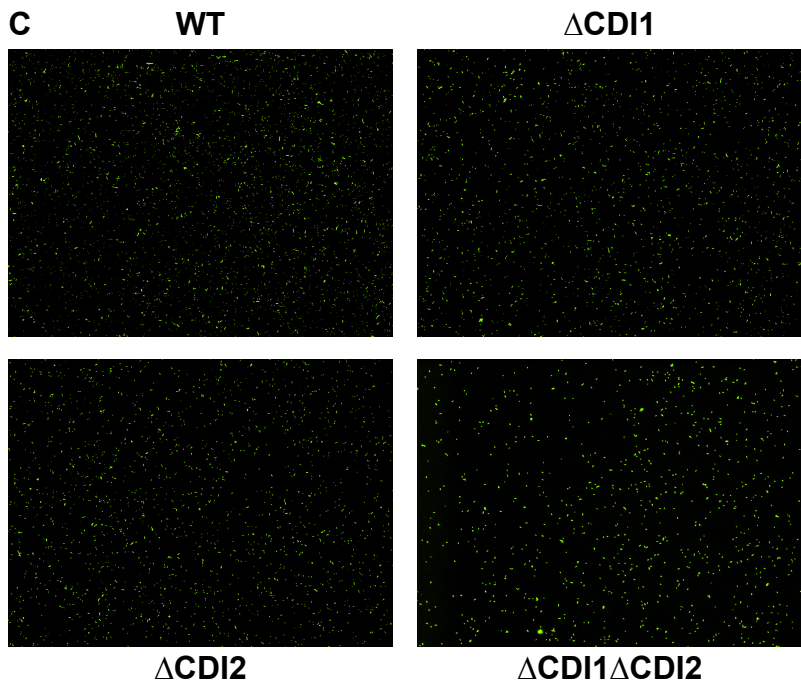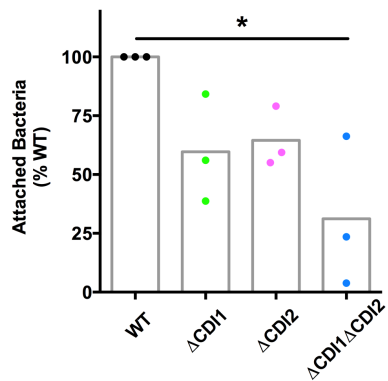

Supplement: FIG S4 [file sph006172403sf4.pdf]

**A**

24 h

72 h

WT

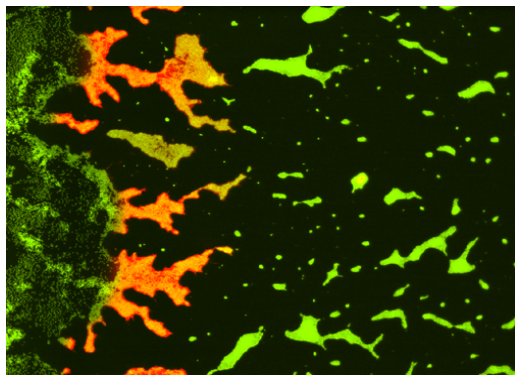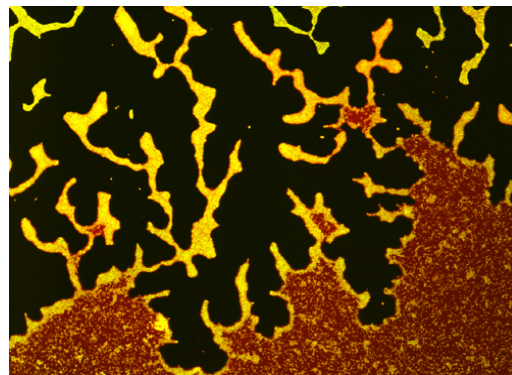 $\Delta$ CDI1 $\Delta$ CDI2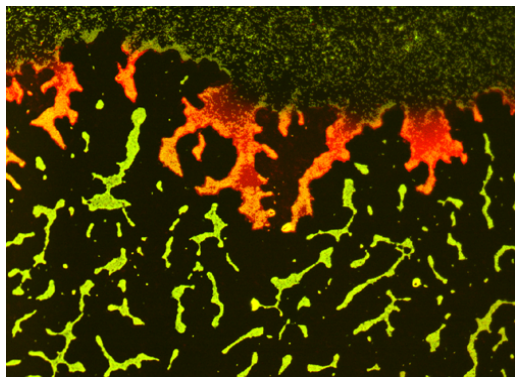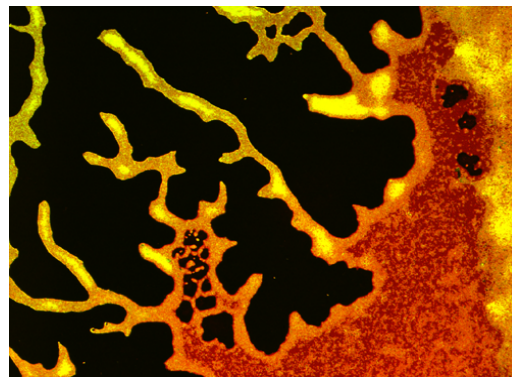

**Green:** *P. aeruginosa*  
**Red:** Psl polysaccharide

**B**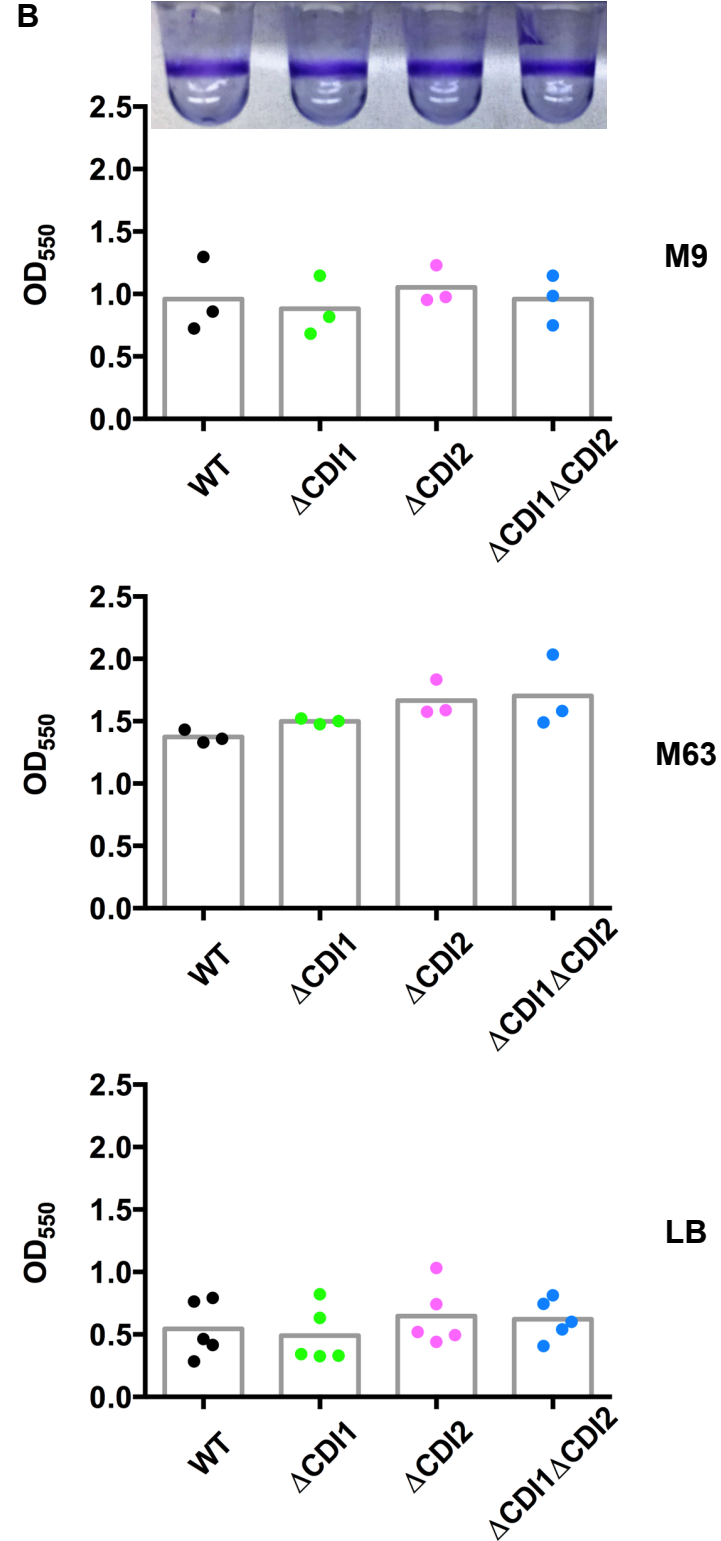

Supplement: FIG S5 [file sph006172403sf5.pdf]

A

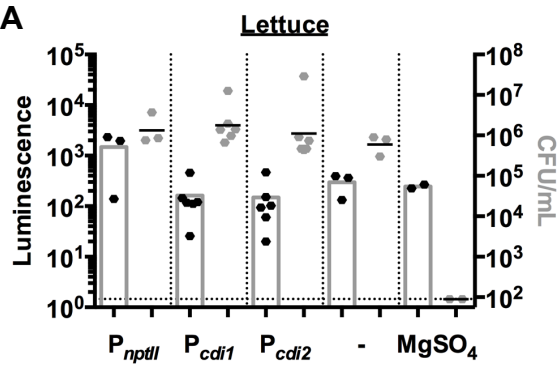

B

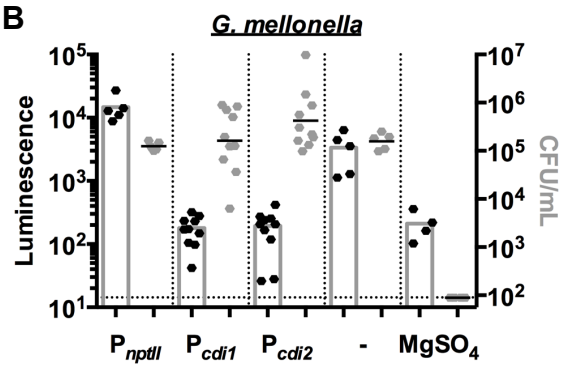

C

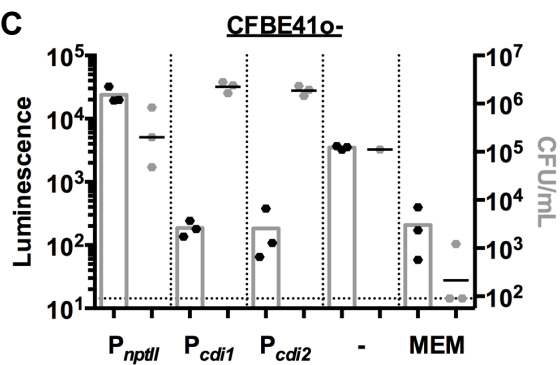

Supplement: FIG S6 [file sph006172403sf6.pdf]

**A**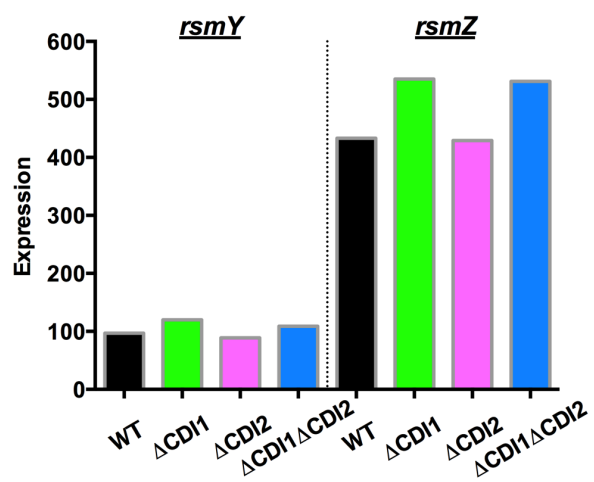**B**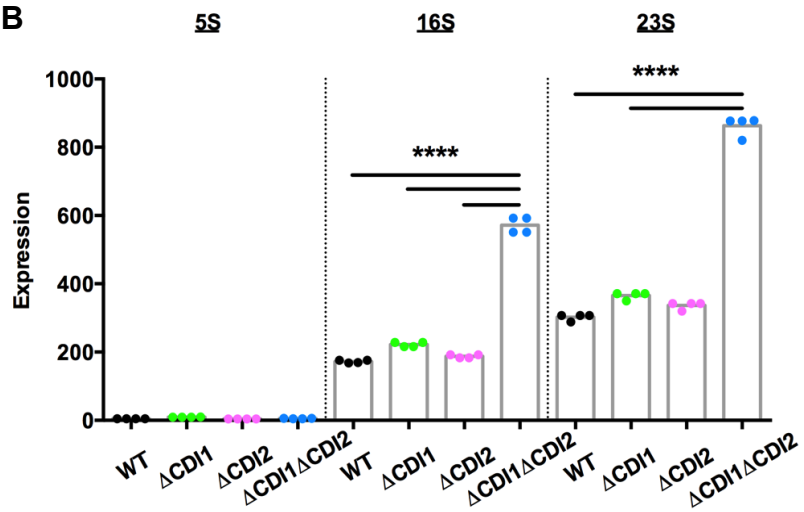

Supplement: FIG S7 [file sph006172403sf7.pdf]
