# Supplementary material for: Pseudomonas aeruginosa Contact-Dependent Growth Inhibition Plays Dual Role in Host-Pathogen Interactions
Source: mSphere. 2017 Nov 15;2(6):e00336-17. doi: 10.1128/mSphere.00336-17 (PMC5687917; doi:10.1128/mSphere.00336-17)
Supplement: TABLE S1 [file sph006172403st8.docx]

**Supplemental Table S1**

|  |  | **Fold change** | | | **q value** | | |
| --- | --- | --- | --- | --- | --- | --- | --- |
| **Gene** | **Gene product** | WT-∆CDI1 | WT-∆CDI2 | WT-∆CDI1∆CDI2 | WT-∆CDI1 | WT-∆CDI2 | WT-∆CDI1∆CDI2 |
|  |  |  |  |  |  |  |  |
| **∆CDI1** |  |  |  |  |  |  |  |
| PA4658 | Hypothetical protein | 0.58 | 0.55 | 0.55 | 2.60E-04 | 2.98E-04 | 0.02 |
|  |  |  |  |  |  |  |  |
| **∆CDI2** |  |  |  |  |  |  |  |
| PA4581.1 | Arg tRNA | 1.16 | 0.34 | 0.33 | 0.90 | 3.31E-32 | 1.96E-16 |
| PA4726.2 | P30 sRNA | 1.72 | 2.66 | 3.34 | 0.90 | 0.03 | 9.68E-08 |
| PA0826.2 | ssrA | 1.02 | 0.86 | 0.99 | 1.00 | 0.01 | 0.03 |
|  |  |  |  |  |  |  |  |
| **∆CDI1∆CDI2** |  |  |  |  |  |  |  |
| PA4280.5 | 16S rRNA | 1.30 | 1.09 | 3.36 | 1.00 | 1.00 | 5.43E-08 |
| PA0668.1 | 16S rRNA | 1.30 | 1.09 | 3.36 | 1.00 | 1.00 | 5.43E-08 |
| PA4690.5 | 16S rRNA | 1.29 | 1.09 | 3.28 | 1.00 | 1.00 | 1.57E-07 |
| PA5369.5 | 16S rRNA | 1.28 | 1.08 | 3.26 | 1.00 | 1.00 | 1.63E-07 |
| PA0668.4 | 23S rRNA | 1.21 | 1.11 | 2.86 | 1.00 | 1.00 | 2.69E-04 |
| PA4690.2 | 23S rRNA | 1.21 | 1.11 | 2.86 | 1.00 | 1.00 | 2.69E-04 |
| PA5369.2 | 23S rRNA | 1.21 | 1.11 | 2.86 | 1.00 | 1.00 | 2.69E-04 |
| PA4280.2 | 23S rRNA | 1.22 | 1.11 | 2.85 | 1.00 | 1.00 | 2.81E-04 |
| PA4421.1 | RNase P RNA subunit | 1.83 | 2.05 | 2.43 | 0.45 | 0.32 | 0.05 |
