# Supplementary material for: Pseudomonas aeruginosa Contact-Dependent Growth Inhibition Plays Dual Role in Host-Pathogen Interactions
Source: mSphere. 2017 Nov 15;2(6):e00336-17. doi: 10.1128/mSphere.00336-17 (PMC5687917; doi:10.1128/mSphere.00336-17)
Supplement: TABLE S2 [file sph006172403st9.docx]

**Supplemental Table S2**

| Primer | Sequence^1^ | Restriction enzyme |
| --- | --- | --- |
| dCDI15F | 5’-GCGCGAGCTC**GTGTTCATGAAGAAGTTGATGTTGGC**-3’ | SacI |
| dCDI15R2 | 5’-GCGCGCTAGC**GATTTCATTCCTTGAAATACAGAAAGGC**-3’ | NheI |
| dCDI13F2 | 5’-GCGCGCTAGC**GCTTTGCTGCGAGAGCCTGAG**-3’ | NheI |
| dCDI13R | 5’-GCGCGGATCC**GGACATGGTTGGTCATCAGGC**-3’ | BamHI |
| dCDI25F | 5’-GCGCGAGCTC**CCGTGCGCCTGATCAACC**-3’ | SacI |
| dCDI25R2 | 5’-GCGCGCTAGC**CCGATGTCCTGCGAGTTCCC**-3’ | NheI |
| dCDI23F2 | 5’-GCGCGCTAGC**TTTTTAAAACATACATCGTTTGCTTTTTC**-3’ | NheI |
| dCDI23R | 5’-GCGCGGATCC**CTTGCATTCATAAGCGCTGCAC**-3’ | BamHI |
| nptIIF2 | 5’-GCGCGAGCTC**ACGCTGCCGCAAGCACTCAG**-3’ | SacI |
| nptIIR2 | 5’-GCGCACTAGT**TCCTCATCCTGTCTCTTGATCAGATCTTG**-3’ | SpeI |
| cdi1IcF3 | 5’-GCGCACTAGT**ATGCCGAAGTACACGTGTACCTGTG**-3’ | SpeI |
| cdi1IcR | 5-‘GCGCGGGCCC**TTACTCAACTATATAAGTGGTGAATCTATTTGGC**-3’ | ApaI |
| cdi2IcF3 | 5’-GCGCACTAGT**ATGTACGCTGATCGTATTGTCAAATTTG**-3’ | SpeI |
| cdi2IcR | 5’-GCGCGGGCCC**CTACGACTTCAAACCCTTCAAGTGTTTG**-3’ | ApaI |
| CDI2IApaF | 5’-GCGCGGGCCC**ATGTACGCTGATCGTATTGTCAAATTTGG**-3’ | ApaI |
| CDI2IKpnR | 5’-GCGCGGTACC**CTACGACTTCAAACCCTTCAAGTGTTTG**-3’ | KpnI |
| Pcdi1FL | 5’-GCGCACTAGT**TGCAGCGCCTGCGACG**-3’ | SpeI |
| Pcdi1RL | 5’-GCGCCTGCAG**GATTTCATTCCTTGAAATACAGAAAGGC**-3’ | PstI |
| Pcdi2FL | 5’-GCGCACTAGT**ATTCGAGTCTGCGTCAAAGCAGC**-3’ | SpeI |
| Pcdi2RL | 5’-GCGCCTGCAG**CCGATGTCCTGCGAGTTCCC**-3’ | PstI |
| cdi12BAF | 5’-**CCCGATCTATTTCCGGGTCGACGCGTTC**-3’ |  |
| cdi12BAR | 5’-**CACTGGTTCAGCGGGCTGCGGATGTCC**-3’ |  |

^1^Underlines indicate restriction enzyme recognition sites engineered into primers for digestion and ligation cloning procedures, bold indicates regions with homology to genome
